# GABA concentrations in the anterior temporal lobe predict human semantic processing

**DOI:** 10.1038/s41598-017-15981-7

**Published:** 2017-11-16

**Authors:** JeYoung Jung, Stephen R. Williams, Faezeh Sanaei Nezhad, Matthew A. Lambon Ralph

**Affiliations:** 1 Neuroscience and Aphasia Research Unit (NARU), Division of Neuroscience & Experimental Psychology, School of Biological Sciences, University of Manchester, Manchester, UK; 2 Centre for Imaging Science and Manchester Academic Health Sciences Centre, University of Manchester, Manchester, UK

## Abstract

There is now considerable convergent evidence from multiple methodologies and clinical studies that the human anterior temporal lobe (ATL) is a semantic representational hub. However, the neurochemical nature of the ATL in the semantic processing remains unclear. The current study investigated the neurochemical mechanism underlying semantic processing in the ATL. We combined functional magnetic resonance imaging (fMRI) with resting-state magnetic resonance spectroscopy (MRS) to measure task-related blood-oxygen level-dependent (BOLD) signal changes during sematic processing and resting-state GABA concentrations in the ATL. Our combined fMRI and MRS investigation showed that the stronger ATL BOLD response induced by the semantic task, the lower GABA concentration in the same region. Moreover, individuals with higher GABA concentration in the ATL showed better semantic performance and stronger BOLD-related fluctuations in the semantic network. Our data demonstrated that the resting-state GABA concentration predicts neural changes in the human ATL and task performance during semantic processing. Our findings indicate that individuals with higher GABA may have a more efficient semantic processing leading to better task performance and imply that GABAergic neurochemical processes are potentially crucial to the neurobiological contribution of the ATL to semantic cognition.

## Introduction

Semantic representation is our collective knowledge about world including words, pictures, objects, people and emotions and its neural basis reflects a large scale network of distributed and interconnected brain regions^1,2^. Converging evidence has shown that the ATL provides a transmodal semantic hub by forming coherent, generalizable semantic representation through the interaction with distributed brain regions^3–11^. Recent distortion-corrected fMRI studies demonstrated increased BOLD responses in the ATL across a range of semantic tasks, regardless of the modality of input^4,5,12,13^. Although there are many investigations of the neural basis of semantic representation and the role of the ATL, the underlying neurochemical mechanisms of the ATL still remain unclear.

Neural activity in the brain is constituted to a large degree by the excitation-inhibition balance, which is closely related to the activities of glutamate and GABA (γ-aminobutyric acid), as the brain’s main excitatory and inhibitory neurotransmitters^14,15^. Recent neuroimaging studies combining MRS and fMRI have demonstrated that the resting-state GABA concentration in various cortical regions (e.g., medial prefrontal cortex, dorsolateral prefrontal cortex, anterior cingulate cortex, insular, visual cortex, and motor cortex) is associated with local stimulus-induced BOLD signal changes as well as inter-regional activity (e.g., spontaneous fluctuation and/or the pattern of coactivation between the functional time-series of brain regions – functional connectivity)^16^. It has been reported that the GABA concentration in the motor cortex is negatively correlated with task-induced BOLD signal changes^17,18^. Within the default mode network (DMN), for example, regional GABA levels in the posterior medial cortex were negatively correlated with BOLD responses and the functional connectivity within the DMN^19^. Another study demonstrated that the regional GABA levels in the striatum were positively correlated with the spontaneous fluctuation within the basal ganglia network, predicting cognitive control processing^20^. These studies suggest that local GABA concentrations predict the amount of BOLD responses in the same region and the connectivity within the network where GABA is measured. Also, GABAergic activity reflected in the beta and gamma frequency bands^21–23^ is involved in various cognitive functions including sensorimotor processing, perception, consciousness, and memory^24–26^. Taken together, these studies imply the potential role of regional GABA in modulating neural activity and cognitive functions in human brain. Therefore, it is possible that there is an interrelation between the ATL GABA levels, ATL BOLD signal changes, functional connectivity and semantic processing. However, it has not been examined until now.

**Figure 1:**
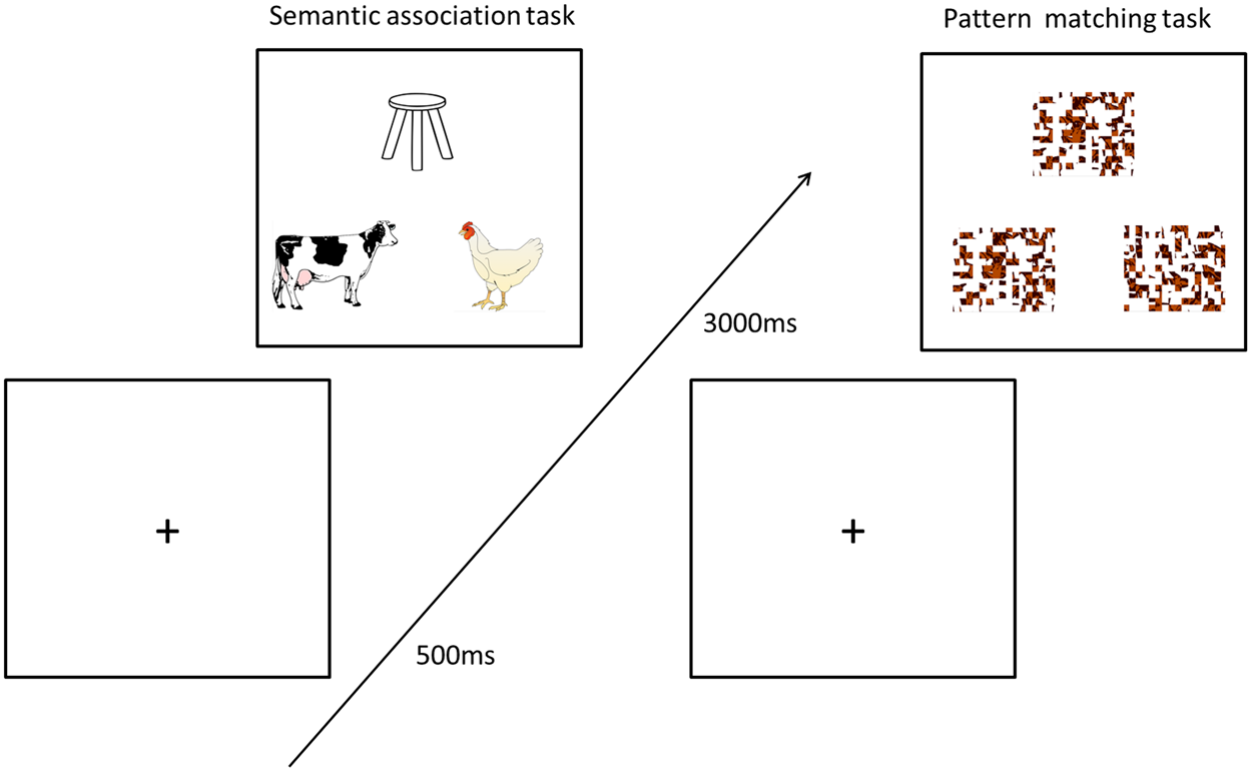
The sequence of a trial of semantic association task and pattern matching task (control task). Each trials consisted of a fixation cross (500 ms) followed by the stimuli. In a trial, three pictures were presented on the screen, a probe picture on the top, the target, and the unrelated picture at the bottom.

Here, we investigate the neurochemical nature of the ATL in semantic processing in 20 healthy participants. We combined fMRI with resting-state MRS to measure the semantic task-related BOLD signal changes and GABA in the left ATL and in the occipital cortex (OCC) as a control region. We applied a GABA-edited MEGA-PRESS spectroscopic sequence to quantify GABA concentrations at rest. Participants performed a semantic association task and a pattern matching task (control task) during fMRI (Fig. 1). BOLD signal changes were measured according to task conditions and the BOLD related fluctuation for semantic processing was determined using an independent component analysis (ICA) approach. The current study investigated whether the steady state GABA concentration in the ATL is associated with BOLD signal changes in the same region as well as BOLD-related network fluctuations during semantic processing. In relation to cognition, it has been reported that resting-state GABA concentration is associated with various cognitive functions^16^. For example, GABA in the primary motor cortex showed a negative correlation with a simple motor execution task^27^, whereas sensory cortex GABA was positively correlated with a perception task requiring fine tactile discrimination^28^ supporting the hypothesis that cortical GABA sharpens cortical activity^14^. In addition, investigations of brain regions related to higher cognition such as cognitive control, motor control and inhibition, have demonstrated that higher GABA in these regions is associated with better task performance^29–32^. Therefore, we hypothesized that (1) as the major inhibitory neurotransmitter, GABA in the ATL would be negatively associated with task-induced BOLD changes in the same region and (2) higher levels of ATL GABA would be beneficial for human semantic processing.

## Results

### Regional analysis

**Figure 2:**
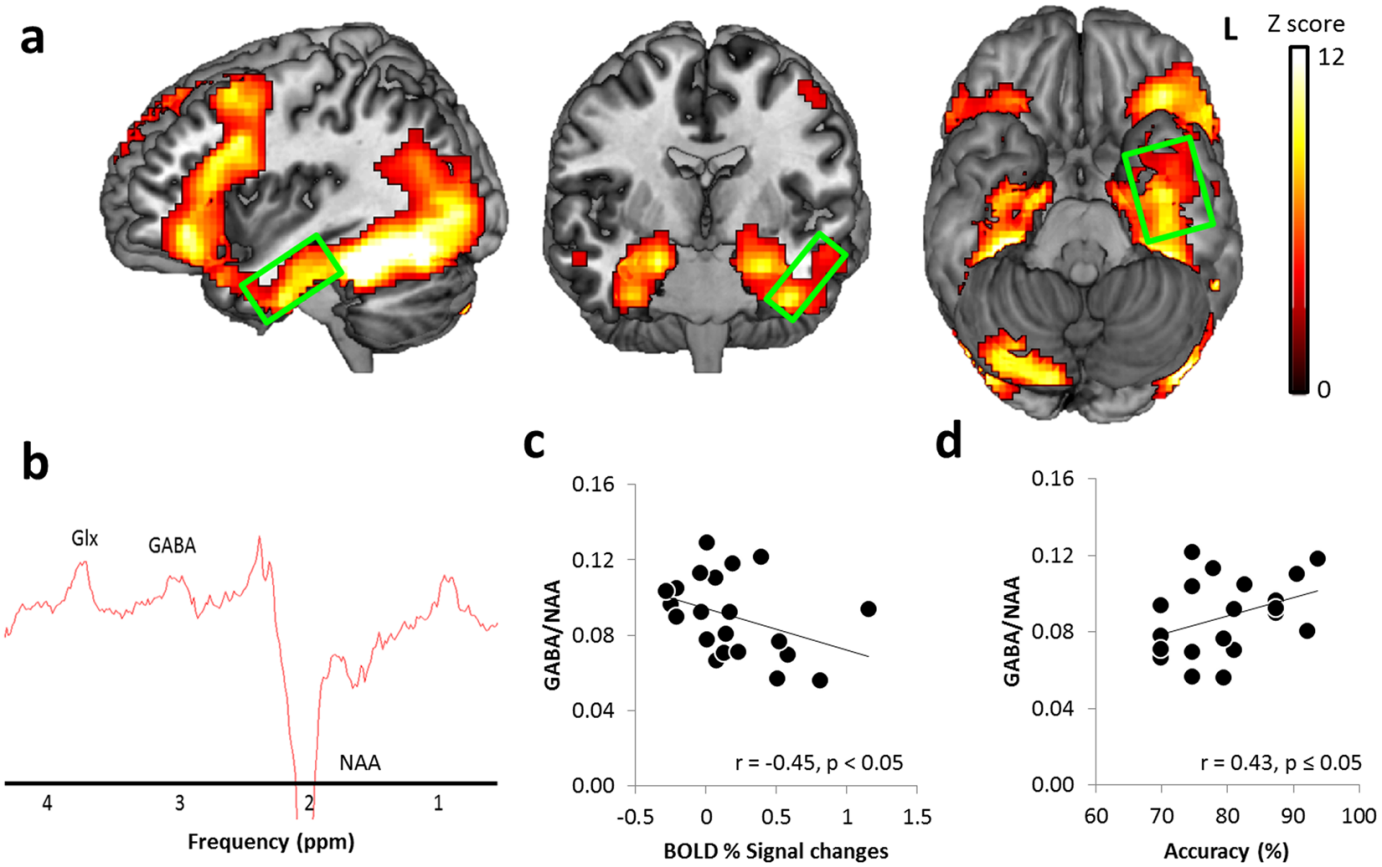
Results of the Brain Imaging Analysis: (**a**) The fMRI BOLD signal associated with a semantic task (semantic > control, thresholded at p FWE-corrected < 0.05, ks > 100). The green box represents the MRS volume of interest in the left anterior temporal lobe. (**b**) A representative MR Spectrum in the ATL with estimated peaks including Glx (a composite measure of glutamate and glutamine), GABA and NAA (N-acetylaspartate). (**c**) Scatterplot showing the negative correlation between individual GABA/NAA ratios and fMRI BOLD signal changes in the ATL. (**d**) Scatterplot showing the positive correlation between individual GABA concentrations and semantic task performance.

The semantic association task evoked increased BOLD signals in the ATL, frontal and posterior temporal cortex (Fig. 2a and Supplementary Table 1) when compared to the control task. GABA and N-acetylaspartate (NAA) as the reference metabolite were quantified for each individual and a ratio between them was used as the regional GABA concentrations (Fig. 2b). Partial correlation analyses accounting for partial volume effects demonstrated that the BOLD signal changes in the ATL were significantly negatively correlated with GABA concentration (p _FDR-corrected_ = 0.039) (Fig. 2c). We observed that, in the ATL, stronger semantic-task BOLD responses were associated with lower GABA concentrations. In contrast, there were no correlations between the ATL GABA and signal changes during the control task and between the OCC GABA and signal changes for the both conditions (ps > 0.41) (Table 1). The control task induced activation in bilateral parietal cortex, posterior fusiform gyrus, and middle/inferior occipital cortex (Supplementary Fig. S1). As there was no significant activation within the OCC volume of interest (VOI) during the control task, we found no significant correlation between the OCC and BOLD signal changes. Also, we compared correlations between the ATL GABA levels and task-induced BOLD signal changes (semantic vs. control). The comparison demonstrated the correlation was significantly greater for the semantic than control task (z = −3.76, p < 0.0001). Our findings suggest task-and regional-specific GABA effects on semantic processing.

**Table 1:** Correlations of neural measurements with GABA concentrations in ROIs.

Neural measurements	Condition	GABA/NAA	
		ATL	OCC
Task-induced ATL signal changes	Semantic	−0.45^*^	−0.11
	Control	0.21	−0.13
Task-induced LFPP		0.35†	−0.17

^*^P fdr-corrected < 0.05, ^†^P fdr-corrected = 0.08.

**Figure 3:**
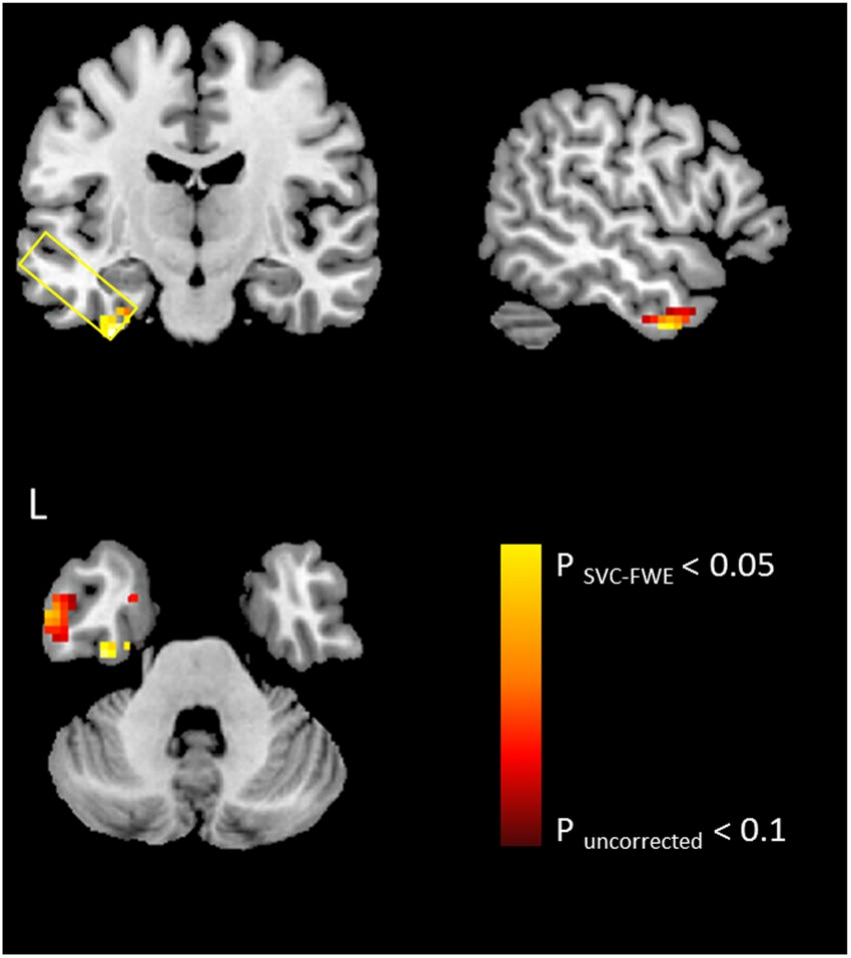
Local maxima of the voxel-wise simple regression analysis of the contrast (semantic > control) with GABA levels in the ATL. The yellow box indicates the size and location of the ATL VOI. The ATL BOLD responses induced by semantic processing correlating with GABA concentrations are restricted to the ventrolateral ATL grey matter even on an uncorrected level of significance (p < 0.1).

To confirm this correlational finding, we carried out a single-voxel regression analysis with the individual’s GABA level in the ATL as the regressor in the fMRI design matrix for the contrast of interest. We observed that BOLD responses in the lateral and ventral ATL were significantly correlated with the GABA levels (MNI −27 −18 −30 and −54 −3 −36, p _SVC-FWE_ < 0.05) and the peak voxels were restricted to grey matter (GM) within the ATL VOI (Fig. 3). Furthermore, a significant positive correlation was observed between resting-state ATL GABA concentrations and semantic task performance (p _FDR-corrected_ = 0.05) (Fig. 2d **and** Table 2). Individuals with higher GABA concentrations in the ATL showed better semantic performance (higher accuracy).

**Table 2:** Correlations of task performance (accuracy) with GABA concentrations in ROIs.

Task performance	ATL	OCC
Semantic task	0.43^*^	−0.07
Control task	0.31	−0.1

^*^p _FDR-corrected_ ≤ 0.05.

### Network analysis

**Figure 4:**
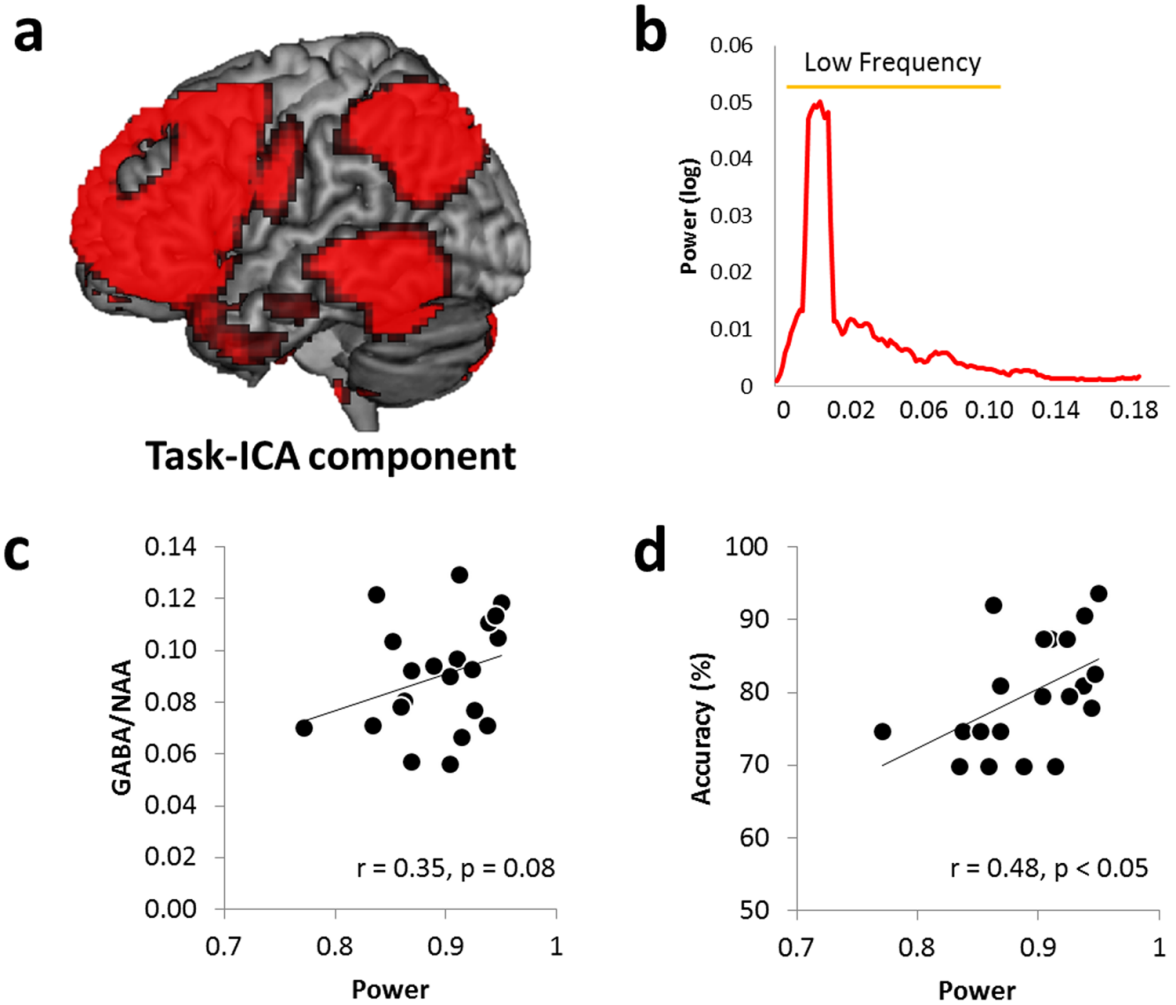
(**a**) Task-ICA component for semantic processing. (**b**) Power of the low frequency range (0.01–0.1 Hz) in the task-ICA component. (**c**) Scatterplot showing the positive correlation between the sum of LFFP and individual GABA concentrations in the ATL. (**d**) Scatterplot showing the positive correlation between the sum of LFFP and individual semantic task performance (accuracy).

The semantic network identified by ICA included the ATL, frontal, parietal, and posterior temporal cortices (Fig. 4a). The sum of low frequency fluctuation power (LFFP) was used to indicate the strength of the signal in this network of interest (Fig. 4b). We found a positive trend between the GABA concentration in the ATL and LFFP of the semantic network (Fig. 4c). Higher ATL GABA concentrations predicted stronger BOLD-related fluctuations in the semantic network. No significant correlation was found between the control region GABA and semantic network LFFP (Table 1). Also, we observed that individuals showing better semantic performance had higher LFFP in the semantic network (Fig. 4d). To confirm these findings, we also examine the relationship between semantic processing and a task-positive network, as a control network. The task-positive network is also called as multiple demand (MD) network, a network of a specific subset of frontal and parietal cortical regions that typically shows increased activity during attention-demanding cognitive tasks^33,34^. The key regions of the network include the dorsolateral prefrontal cortex, inferior frontal gyrus, insular, pre-supplementary motor area, anterior cingulate cortex, and intraparietal sulcus. Activity increases in these regions are generally proportional to task difficulty. Therefore, we correlated the ATL GABA concentrations with the MD network in order to demonstrate that the ATL GABA is specifically associated with semantic processing. The results showed that there was no significant correlation between the ATL GABA and the MD network (p = 0.2) (Supplementary Fig. S2). We also compared two correlations (ATL GABA-semantic network vs. ATL GABA- MD network) and showed a marginally significant result (z-score = 1.49, p = 0.067).

As all measurements (the ATL GABA concentrations, semantic task-induced ATL activity, semantic network LFFP, and semantic task performance) were inter-correlated with each other, we conducted partial correlation analyses, by regressing out other related variables in order to confirm our key findings. The results demonstrated that the ATL GABA was significantly associated with the ATL regional activity, regressing out the semantic task performance, semantic network LFFP and GM volume (r = −0.47, p = 0.024). Also, the ATL GABA correlated with the semantic task performance, regressing out the ATL regional activity, semantic network LFFP, and GM volume (r = 0.42, p = 0.044).

### Best predictors of task accuracy

To examine these correlational findings in more detail and to explain the highest amount of task-accuracy variance, we carried out a multiple regression analysis, by modelling the GABA concentration (β = 0.18, p < 0.05), regional signal change in the ATL (β = −0.15, p = 0.058), LFFP (β = 0.25, p < 0.05), and age (β = 0.62, p < 0.001). The full model explained 63% of the task-accuracy variance (adjusted *R* ^2^) and was highly significant (F_4_, _15_ = 9.00, p < 0.001). In the absence of age, fMRI and MRS measurements accounted for 29% of the variance in accuracy (F_3_, _16_ = 3.55, p = 0.05).

## Discussion

Accumulating and converging evidence has implicated the ATL as a transmodal hub for semantic representation^11,35^ but the underlying neurochemical mechanism of the ATL remains unclear. Here, we investigated this important issue by combining fMRI and MRS. Our combined fMRI and MRS investigation demonstrated that the ATL GABA concentrations predict semantic performance and neural changes during semantic processing. Our findings suggest that ATL GABA neurobiology may play a potential role in modulating neural mechanisms underlying semantic processing.

To our best knowledge, this is the first study demonstrated that the resting-state GABA concentration predicts neural changes in the ATL and task performance during semantic processing. As a major inhibitory neurotransmitter, GABA is negatively coupled with regional BOLD changes^27,36^. Here, we found that higher ATL GABA concentrations were associated with lower BOLD signal changes in the same region as well as better semantic performance. Semantic processing allows us to produce time- and context- appropriate behaviours. For example, when we see a milking stool (Fig. 1), all information related to the concept is activated in the semantic representation (e.g., wood, brown, farm, cow, milk, sitting and etc). In order to choose the correct response (cow), irrelevant meanings need to be suppressed a process that requires an ‘inhibitory’ mechanism. Thus, if individuals have relatively higher GABA levels in the ATL semantic hub, this inhibitory processing may be more efficient and effective. A related idea has been proposed previously in the context of the close yoking of activation and inhibition in early visual areas^37^. In the healthy visual system the rise of excitatory glutamate is followed closely by upregulation of inhibitory GABA. When these paired excitatory-inhibitory functions are tightly coupled then there is a resultant sharpening of the activated representation^14^. It is easy to imagine that a similar process may operate in higher order association cortices including the ATL semantic hub – thus leading to more sharply defined semantic representations and thus more accurate performance. Also, our finding that lower task-induced ATL activity was associated with better semantic performance supports the neural efficiency hypothesis^38^: that better task performance is associated with less brain activation. Overall, our results suggest that higher levels of local GABA may help to sharpen activated distributed semantic representations through lateral inhibition^39^.

Recent MRS combined with fMRI studies have demonstrated that neural changes including the regional BOLD and network-level changes are associated with GABA concentrations in other brain regions^14,16,27^. We found a positive trend between the ATL GABA levels and the strength of semantic network (p _FDR-corrected_ = 0.08). This finding is in line with pharmacological studies on resting-state brain activity in healthy participants. Pharmacological intervention investigations have demonstrated that GABA agonists increased BOLD synchrony (strength of network/functional connectivity) in multiple resting-state networks with midazolam^40,41^ and zolpidem^25^. GABA modulators such as zolpidem and benzodiazepines have a specific effects on EEG frequency bands; they decreases alpha (8~12 Hz) power, whereas increases power in the beta (13~30 Hz) and gamma (>30 Hz)^21,42,43^. It has been shown that GABAergic activity coupled with the beta and gamma rhythms^22,23^ are involved in various functions including sensorimotor, perception, memory and consciousness^24–26^. Especially, as GABAergic neurons are prone to synchronize in the gamma frequency bandwidth, the increased network connectivity by GABAergic drugs potentially reflect the enhanced synchrony among neurons across the brain^44^. Fries^45^ suggested that synchrony can be an efficient and precise means for controlling information processing by enhancing salience and local communication within connected networks. Here, we found that individuals with stronger power in the semantic network showed better task performance and higher GABA concentration in the ATL predicted better semantic performance. These findings suggest that higher GABA concentration in the ATL may lead to more efficient semantic processing. Studies on other cognitive domains such as inhibition and cognitive control had been shown that higher GABA concentrations are related to better task performance^20,29–32,46^. These studies suggest that higher GABA can be beneficial for higher human cognition such as inhibitory control processing.

Our analysis showed that the ATL GABA levels, ATL task-induced activity and task-related network strength have a direct relationship with task performance. Better semantic processing was associated with both higher ATL GABA and stronger semantic network power as well as less regional ATL activity during semantic processing. This finding suggests that multiple factors contribute to semantic performance including the level of ATL GABA.

It should be noted that all variables measured in this study (the ATL GABA concentrations, task-induced ATL activity, semantic network LFFP, semantic task performance) were inter-correlated with each other (ps < 0.05). However, our regression analyses demonstrated that the ATL GABA levels were significantly associated with task-induced regional activity (p _SVC-FWE_ < 0.05, Fig. 3). Also, we had a control VOI (the occipital cortex) and a control task. Our results showed that there was no significant correlation found in the control VOI and the control task. This suggests that our findings were task-and regional-specific. To confirm this interpretation, we compared correlations between the ATL GABA with regional activity during semantic processing and control processing (pattern matching). The comparison demonstrated that the relationship between the ATL GABA and ATL regional activity was significantly greater for the semantic than control task. In addition, partial correlation analyses were performed, regressing out other related variables in order to confirm our key semantic interpretation of the results. These additional analyses demonstrated that ATL GABA was significantly associated with ATL regional activity, even after accounting for the GM volume, semantic task performance and semantic network LFFP. Likewise, ATL GABA was correlated with semantic task performance, even after accounting for the GM volume, ATL regional activity and semantic network LFFP. In short, these additional analyses support our key proposition that the regional ATL GABA plays a critical role in shaping its neural activity involved in semantic processing.

In conclusion, we demonstrated that resting-state ATL GABA concentration predicts the strength of neural responses during semantic processing. Moreover, our data indicate that individuals with higher GABA have more efficient cognitive processing and network function leading to better task performance. These findings suggest the GABAergic action in the ATL shapes semantic processing.

## Materials and Methods

### Participants

We recruited twenty healthy English native speakers (7 males, mean age = 23 years ± 4, range from 20 to 36 years). All participants were right-handed as assessed by the Edinburgh Inventory for Handedness^47^. After a detailed explanation of the study, all participants gave their written informed consent. The experiment was approved by the ethics committee of the University of Manchester in accordance with the Declaration of Helsinki.

### Experimental design and procedure

All participants performed a semantic association task and a pattern matching task (control task) during fMRI and again outside of the scanner on different days. The semantic association task was adapted from previous rTMS and fMRI studies^5,48^. The items for the semantic association task were created by combining the Pyramids and Palm Trees test (PPT)^49^ and an abridged version of the Camel and Cactus test (CCT)^50^. The items for the pattern matching task were created by scrambling the pictures used in the semantic association task. The semantic association task required participants to select which of two pictures was more related in meaning to a probe picture. In each trial, three pictures were presented on the screen, a probe picture on the top, the target, and the unrelated picture at the bottom (Fig. 1 left). The pattern matching task asked participants to choose which of two patterns was identical to a probe pattern (Fig. 1 right). E-prime software (Psychology Software Tools Inc., Pittsburgh, USA) was used to display stimuli and to record responses.

In the scanner, participants performed an fMRI scanning consisting of 9 blocks of the semantic association task and 9 blocks of the pattern matching task interleaved (e.g., A-B-A-B). Between the task blocks, there were fixation blocks for 4000 ms. A task block consisted of 4 trials of each task. A trial started with 500 ms fixation followed by the stimuli presented for a fixed duration of 4500 ms. The total time of a session was about 7 mins. Participants pressed one of two buttons designating two choices in a trial.

The behavioural performance was measured outside of the scanner. The experiment was conducted within a week after scanning. Participants performed the semantic association task and pattern matching task. Each task had 63 trials and each trial started with 500 ms fixation then the stimuli were presented until response or 3000 ms. The order of trials was randomized. The semantic performance was ACC: 78.8% ± 8.3 ranging from 68% ~ 94% and RT: 2357 ms ± 490 ranging from 1719 ms to 2993 ms and the control task performance was ACC: 91.6% ± 7.2 ranging from 80~100% and RT: 2243 ms ± 372 ranging from 1665 ms to 2859 ms.

### fMRI data acquisition and analysis

Imaging was performed on a 3T Philips Achieva scanner using a 32-channel head coil with a SENSE factor 2.5. To maximise signal-to-noise (SNR) in the ATL, we utilised a dual-echo fMRI protocol developed by Halai *et al*.^51^. The fMRI sequence included 42 slices, 96 × 96 matrix, 240 × 240 × 126 mm FOV, in-plane resolution 2.5 × 2.5, slice thickness 3 mm, TR = 2.8 s, TE = 12 ms and 35 ms. The total volume of scanning was 258. A T1-weighted structural image was acquired using a 3D MPRAGE pulse sequence with 200 slices, in-planed resolution 0.94 × 0.94 mm slice thickness 0.9 mm, TR = 8.4 ms, TE = 3.9 ms.

Image processing and statistical analyses were carried out using MATLAB R2012a and SPM8. The dual gradient echo images were extracted and averaged using in-house MATLAB code developed by Halai *et al*.^51^. Functional images were realigned correcting for motion artefacts and different signal acquisition times by shifting the signal measured in each slice relative to the acquisition of the middle slice prior to combining the short and long echo images. The mean functional EPI image was co-registered to the individual T1-weighted image and segmented using the DARTEL (diffeomorphic anatomical registration through an exponentiated lie algebra) toolbox^52^. Then, normalization was performed using DARTEL to warp and reslice images into MNI space and smoothing was applied with an 8 mm full-width half-maximum Gaussian filter.

Statistical analyses were carried out using a general linear model (GLM). For each participant, we defined a design matrix modelling semantic (semantic association block), control (pattern matching block) and baseline (fixation). Additionally, six motion parameters were included as regressors in the design matrix. Contrast images were computed to assess differences in activations between the semantic and control tasks (semantic association > pattern matching) for each participant. Group analyses were conducted using a random effect model (one-sample t tests). Statistical threshold was set at p < 0.001 at the voxel-level and p < 0.05 at the cluster level with at least 100 contiguous voxels after family-wise error (FWE) correction.

Regions of interest (ROI) analysis was carried out using Marsbar^53^. The mean signal changes of voxels within the ROI (ATL) were extracted for each contrast (semantic, control and fixation). The ATL ROI was defined identically in size and orientation as our ATL VOI of MRS acquisition (averaged across all participants).

### Independent component analysis

For the independent component analysis (ICA), the pre-processed images were entered into the GIFT toolbox^54^. This toolbox provides a group ICA approach, which concatenates the individual data followed by the computation of subject-specific components and time courses. Individual data were reduced by using principal component analysis. Then, informax algorithm was chosen for the group ICA. Using ICASSO, the algorithm was run 20 times to improve the independent components’ (IC) stability. The resultant 32 ICs were visually inspected and then spatially sorted against the brain activation map of the GLM analysis. The selected group ICs (semantic network and multiple demanding network as a control) was back reconstructed using the GICA algorithm to create subject-specific component maps and time courses. The data were entered into spectral analyses using MANCOVAN, which calculate the power of each frequency within the measured frequency band [0–0.2 Hz, given a Nyquist frequency of 1/(TR/2)]. Individual time courses were log-transformed to obtain a normal distribution before the statistical analyses. The sum of low frequency fluctuation power (LFFP, 0.01–0.1 Hz) was used to determine the strength of the signal of interest within the semantic network.

### MRS data acquisition analysis

Single voxel ^1^H MRS data were acquired at rest from two volumes of interest (VOI) in each participant after a T1-weighted structural image acquisition using a 3 T Philips Achieva scanner with a 32-channel head coil. One voxel of 35 × 25 × 15 mm was placed in the left ATL. Given that recent research suggests that the ATL semantic hub is centred on the ventrolateral subregion^11,55^, we ensured that the VOI covered the ventrolateral area (anterior fusiform gyrus and inferior temporal gyrus) and avoided hippocampus. A control VOI (OCC, 30 × 30 × 30 mm) was placed within the posterior occipital lobe, centred on the mid-sagittal plane to cover both hemispheres (Supplementary Fig. S3). For detection of GABA, GABA-edited MEGA-PRESS spectra were acquired with a repetition time = 2000 ms, echo time = 68 ms. Spectra were acquired in interleaved blocks of 4 scans with application of the MEGA inversion pulses at 1.95 ppm (79 repeats at the ATL VOI and 74 repeats at the OCC VOI). It is noted that the ATL is prone to magnetic susceptibility artifacts. In order to overcome this difficulty, we tested several different protocols and adapted the MEGA-PRESS sequence for the current study. A total of 1024 sample points were collected at a spectral width of 2 kHz. Each MRS voxel took approximately 10 mins to acquire.

Quantification was conducted using the Advanced Magnetic Resonance (AMARES) routine^56^ in the Java-based magnetic resonance user’s interface (jMRUI5.1, EU project). The water resonance was removed using the Hankel Lanczos Singular Valve Decomposition (HLSVD) algorithm^57^. To improve the display of the spectra, line broadening of 7 Hz was used. No time-domain filtering was performed on the data before analysis by AMARES. All metabolite resonances were measured including GABA, glx and NAA. A ratio was calculated for GABA/NAA and Glx/NAA (i.e., NAA is one of the most concentrated molecules with the largest peak on proton MR spectrograms of healthy human brain and widely used as the reference compound). No correlation of GABA levels between the ATL and OCC was found (p = 0.47). The GABA concentrations of OCC did not correlate with any measurements (BOLD responses, task performance and LFFP) in the study. We also obtained a combined measure of glutamate and glutamine in the ‘Glx’ peak. However, the Glx concentrations (Glx/NAA) in the ATL did not correlate with either the BOLD responses or with GABA (ps > 0.37). The main interest of this study is the role of GABA on semantic processing so we did not investigate Glx any further.

To examine partial volume effects on MRS VOIs, the T1-weighted anatomical images were segmented into gray matter (GM), white matter (WM) and cerebrospinal fluid (CSF) using SPM8. Then voxel registration was performed using custom-made scripts developed in MATLAB by Dr. Nia Goulden, which can be accessed at http://biu.bangor.ac.uk/projects.php.en. The scripts generated a mask for voxel location by combining location information for the Philips SPAR file with orientation and location information contained within the T1 image. The calculation of partial volume within the VOIs provided the percentage of each tissue type within the relevant voxels. The ATL VOI consisted of 51% of GM, 46% of WM, and 2% of CSF and the OCC VOI, 55% of GM, 31% of WM, and 15% of CSF. It should be noted that neurotransmitter concentrations are substantially higher in the GM compared with WM. Previous studies have shown that the GABA concentrations are significantly (two-fold) higher in the GM than WM^58,59^. Therefore, it can be assumed that mainly cortical regions contribute to the observed GABA-BOLD response correlation in the ATL. Since GABA concentration in the voxel will be related to GM content, we tested whether GM itself correlated with BOLD signal or task performance. % GM did not correlate either with semantic performance (r = 0.16, p = 0.50) or with the ATL BOLD responses (r = 0.26, p = 0.26)

### Relationship between MRS, fMRI measurements and behavioural performance

Partial correlation analyses investigated relationships between task performance, GABA concentrations, regional signal change in the ATL, and LFFP of the semantic network, taking the proportion of GM volume within the given MRS VOIs as a covariate. False-discovery rate (FDR) correction was applied for multiple comparisons. We reported the results thresholded at p _FDR-corrected_ < 0.05 (one-tailed). Multiple regression analyses were performed to determine the combination of variables that explained the greatest amount of variance in task accuracy, by modelling the GABA levels, regional signal change in the ATL, LFFP and age.

To account for the comparably large size of our MRS VOI we further tried to reveal local maxima of voxels within the ROI correlating in their BOLD signal with GABA levels. The ATL ROI that showed a significant correlation of the BOLD signal changes with GABA levels was tested voxelwise by performing a standard second level simple regression analysis on subject’s first level contrast image (semantic > control) within the ROI. Local maxima of correlation were estimated on a voxel level setting the threshold to p < 0.05 FWE after small-volume correction.

## Electronic supplementary material

Supplementary Information
