# Supplementary material for: GABA concentrations in the anterior temporal lobe predict human semantic processing
Source: Sci Rep. 2017 Nov 16;7:15748. doi: 10.1038/s41598-017-15981-7 (PMC5691052; doi:10.1038/s41598-017-15981-7)
Supplement: Supplementary file 1 — Supplementary Information [file 41598_2017_15981_MOESM1_ESM.doc]

**GABA concentrations in the anterior temporal lobe predict human semantic processing**

JeYoung Jung, Stephen R. Williams^2^, Faezeh Sanaei Nezhad^2^ & Matthew A. Lambon Ralph

**Supplementary information**

Supplementary Fig.1

Supplementary Fig.2

Supplementary Table 1

**Supplementary Fig.1**


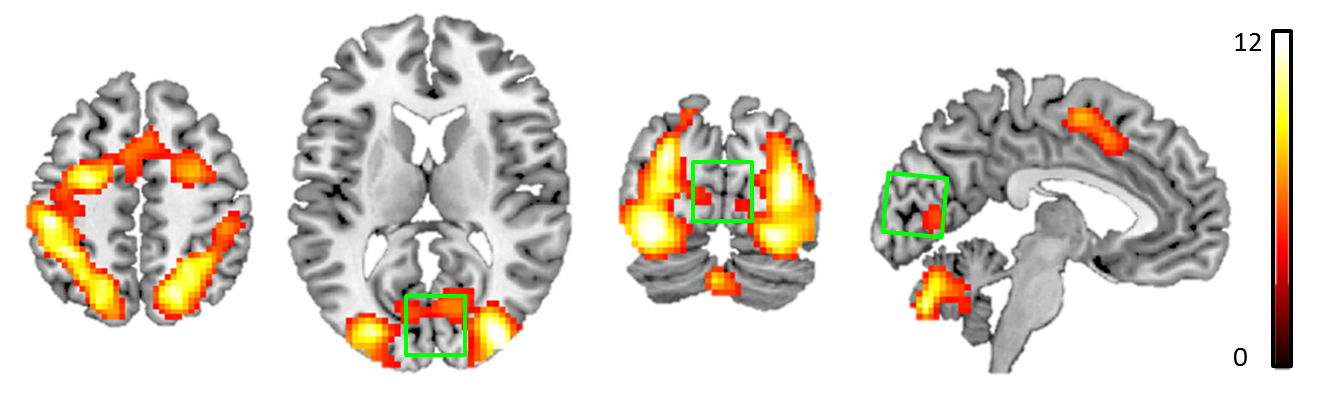


**Figure S1**. Brain activation map (Control task > baseline) and the OCC VOI (green box). The colour bar indicates Z-score.

**Supplementary Fig.2**


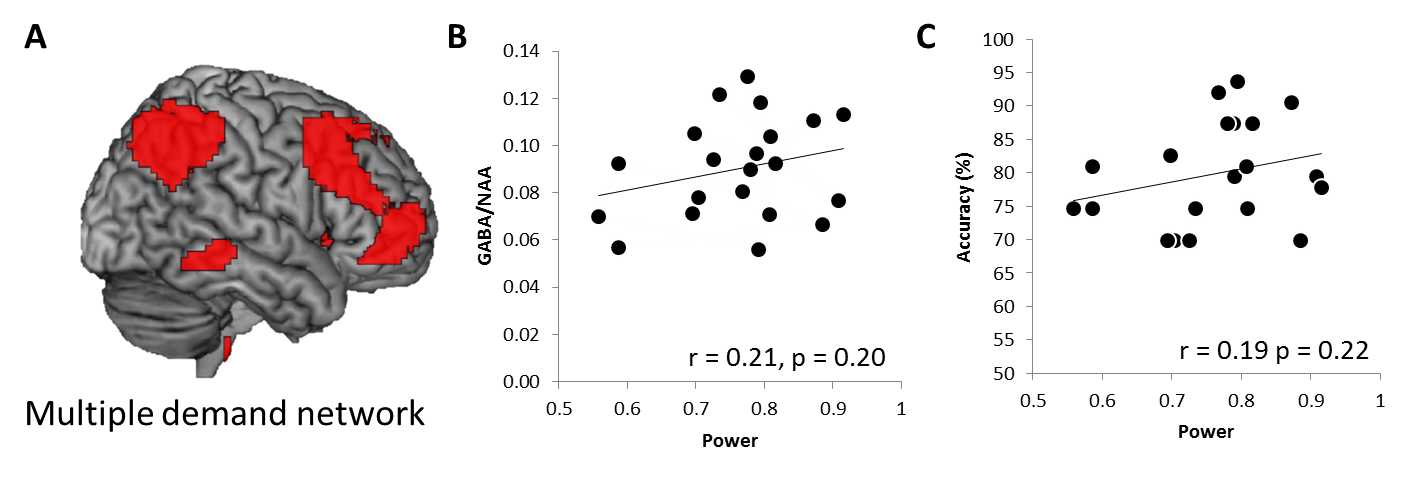


**Figure S2**. A) Task-ICA component for multiple demanding (MD) network. B) No significant correlation between the ATL GABA and MD network power. C) No significant correlation between the MD network power and semantic task accuracy.

**Supplementary Fig. 3**


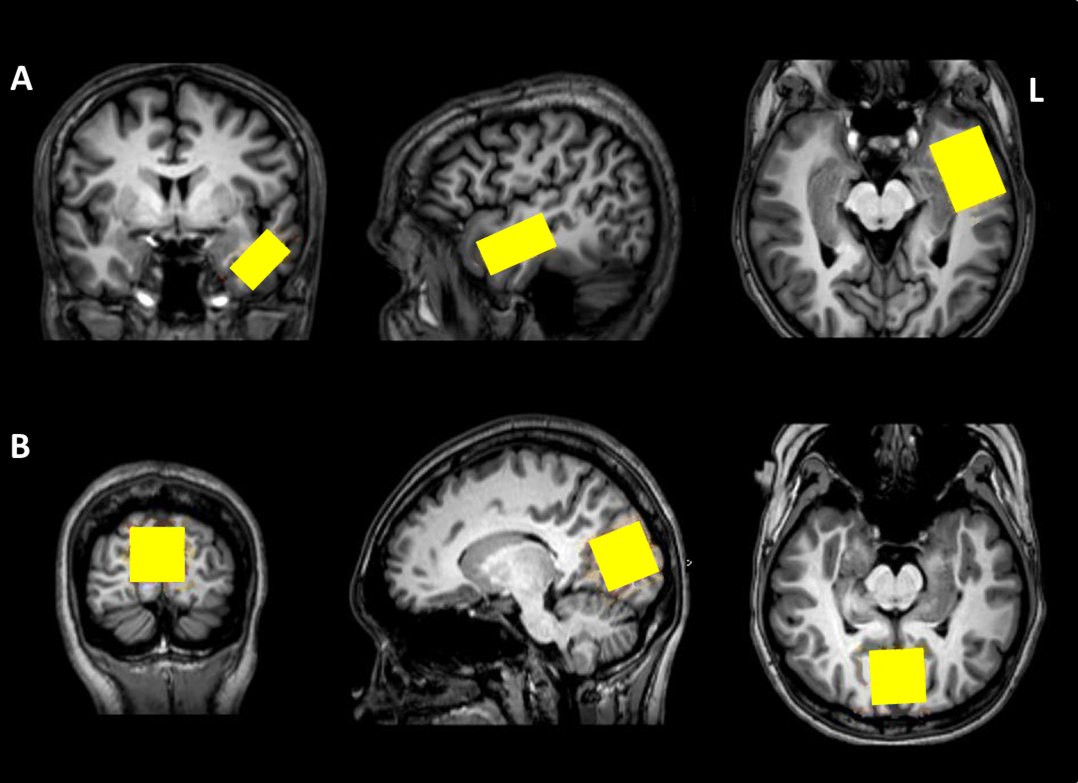


**Figure S3**. The location of MRS VOI. A) ATL VOL. B) OCC VOI (control).

**Supplementary Table 1. Whole brain analysis of the contrast of interest (semantic > control task).**

| Cluster | Brain region | MNI | | |
| --- | --- | --- | --- | --- |
|  |  | x | y | z |
| 6151 | Middle Temporal Gyrus | -57 | -48 | -3 |
|  | Fusiform Gyrus | -39 | -42 | -24 |
|  |  | -39 | -66 | -15 |
|  |  | -39 | -57 | -15 |
|  | Inferior Occipital Gyrus | -42 | -72 | -6 |
|  |  | -42 | -81 | -3 |
|  | Middle Occipital Gyrus | -45 | -78 | 6 |
|  | IFG (p. Triangularis) | -48 | 21 | 24 |
|  |  | -45 | 30 | 15 |
|  |  | -48 | 33 | 12 |
|  | Middle Frontal Gyrus | -45 | 12 | 51 |
| 2777 | Fusiform Gyrus | 36 | -45 | -21 |
|  |  | 39 | -57 | -18 |
|  |  | 27 | -30 | -21 |
|  | Middle Occipital Gyrus | 48 | -75 | 3 |
|  | Inferior Occipital Gyrus | 36 | -66 | -12 |
|  |  | 42 | -81 | -6 |
|  | Hippocampus | 21 | -6 | -21 |
|  |  | 36 | -18 | -18 |
|  | Cerebellum | 30 | -72 | -39 |
|  |  | 15 | -87 | -39 |
| 374 | IFG (p. Triangularis) | 57 | 24 | 21 |
|  |  | 57 | 27 | 3 |
|  |  | 57 | 33 | 12 |
|  | IFG (p. Orbitalis) | 30 | 33 | -15 |
|  |  |  |  |  |
